# A Mixed-Methods Assessment of Health Care Providers' Knowledge, Attitudes, and Practices Around Fertility Awareness-Based Methods in Title X Clinics in the United States

**DOI:** 10.1089/whr.2020.0065

**Published:** 2020-09-15

**Authors:** Shelby Webb, An-Lin Cheng, Rebecca Simmons, Rachel Peragallo Urrutia, Victoria Jennings, Jacki Witt

**Affiliations:** School of Nursing and Health Studies, University of Missouri-Kansas City, Kansas City, Missouri, USA.

**Keywords:** attitudes, awareness, contraception, fertility, provider, Title X

## Abstract

***Objective:*** To understand how Title X providers currently engage with fertility awareness-based methods (FABMs) for pregnancy prevention in Title X clinics across the United States.

***Materials and Methods:*** We developed a survey to assess knowledge of fertility for purposes of pregnancy prevention, attitudes toward FABMs use for pregnancy prevention, and practices when patients request FABMs for pregnancy prevention.

***Results:*** In total, 329 participants who met all inclusion criteria completed the survey. Respondents were generally highly knowledgeable on fertility, felt neutrally toward FABMs or thought they were a nonviable option for most women, and were likely to respond to patient requests for FABMs for pregnancy prevention by providing information. Qualitative responses included several barriers to provision of FABMs for pregnancy prevention and few successes to provision.

***Conclusions:*** Fertility knowledge and discussion of specific methods increased with the number of methods included in the clinic's written materials or with the number of different FABMs someone at that clinic had been trained on. Significant clinician or administrative barriers may exist to offering FABMs to patients. Incorporating up-to-date information on a range of FABMs—rather than treating them as one method—into contraceptive counseling represents an opportunity to increase the contraceptive offering for clients who want them, leading to increased patient satisfaction and successful family planning outcomes.

## Introduction

Fertility awareness-based methods (FABMs) of family planning rely on women interpreting their individual physiological signs, such as the timing of the menstrual cycle, changes in cervical fluid, basal body temperature, and/or increases in urinary hormones to predict current fecundity. The underlying contraceptive principle of FABMs is that people can reduce their chance of pregnancy by abstaining from vaginal intercourse or using an alternative method of contraception during days of potential fecundity.

There are many different FABMs, each relying on different method rules and one or more physiological signs.^1^ For example, the Standard Days method relies only on menstrual cycle timing, and women consider themselves fertile between days 8 and 19, whereas symptothermal methods utilize signs such as cervical fluid and basal body temperature in combination to identify fertile days. A recent systematic review found moderate quality typical use pregnancy estimates for 12 specific FABMs ranging from 2% to 33% per 100 woman/years.^1^

FABMs are the preferred method of use for a small but growing number of contraceptive users of FABMs in the United States.^2^ The Office of Population Affairs reported an increase in the number of female family planning users whose primary method of contraception is FABMs from 8784 in 2007 to 15,287 in 2017.^3,4^ People who use these methods may desire to avoid hormones, adhere to religious teachings, involve the male partner in reproductive decision making, and/or feel more in tune to the functioning of their reproductive system. Besides contraceptive uses, FABMs may also be used by women or couples to achieve a pregnancy or monitor health conditions, such as polycystic ovary syndrome and infertility.^5^ In turn, they must accept that these methods may be less effective than some other methods and are especially susceptible to imperfect use. People who use FABMs deserve transparent information about their effectiveness, benefits, and challenges.

Recently, the Office of Population Affairs, which administers the Title X grant program, identified FABMs as a key topic for the Title X network, with requirements for Title X providers to offer counseling on these methods as part of offering a broad range of contraceptive methods. Despite increasing demand and federal attention, little is known about current provider knowledge, attitudes, and practices related to FABM counseling and provision,^6,7^ and nothing is known about these aspects specific to Title X providers. The purpose of this study was to understand how Title X providers currently engage with FABMs for pregnancy prevention in Title X clinics across the United States.

## Materials and Methods

To assess provider knowledge, attitudes, and practices related to FABMs for pregnancy prevention, we developed a survey for Title X-funded clinic staff across the United States.

### Participants

We posted a link to the survey on the Title X National Clinical Training Center for Family Planning website and additional survey links in newsletters from the Title X Family Planning National Training Center, the Office of Population Affairs, and the National Family Planning and Reproductive Health Association. It was also sent directly to e-mail addresses on file for providers at Title X clinics.

Responses were included in this analysis if respondents affirmed that their primary clinic setting provided family planning services and received federal Title X family planning funds and that they held a clinical role in that setting (nurse, provider, *etc.*). Responses from manager/administrators or billing/finance staff were excluded from this analysis, which was focused on understanding FABM knowledge, attitudes, and practices among providers of contraception and/or contraceptive counseling.

### Instruments

The survey instrument consisted of a 53-item survey created by the authors and tested by three FABM subject matter experts. Both the terms “FABMs” and “NFP” (natural family planning) were defined in the survey to ensure respondents understood that “FABMs” included all fertility tracking methods. Survey items included multiple choice, true/false, yes/no options, Likert-type, and open text questions. The survey questionnaire is provided in Appendix A1.

#### Knowledge

We assessed knowledge of fertility for purposes of pregnancy prevention with four true/false “knowledge” questions. These questions included identification of the normal menstrual cycle length, characteristics of cervical mucus around the time of ovulation, normal basal body temperature increase after ovulation, and length of the luteal phase.

#### Attitudes

To assess provider attitudes toward FABM use for pregnancy prevention, we asked the participants to rate the viability of FABMs as a method of contraception on a Likert scale, with 1 being “a nonviable option for most women,” 3 being “neutral,” and 5 being “a viable option for most women.”

#### Practices

To assess provider behaviors, we asked participants to describe how they responded to patients requesting an FABM for pregnancy prevention. In addition, we asked two open-text questions querying providers about existing barriers to providing FABMs, as well as their experiences of success in providing these methods for pregnancy prevention.

### Data collection

The survey was hosted by REDCap^8^ and contained a consent script that explained the purpose of the study and ensured participation was anonymous and voluntary. The survey was open for 11 weeks. This study was approved as exempt with a waiver of signed informed consent by the University of Missouri-Kansas City Institutional Review Board.

### Data analysis

For quantitative responses, we conducted descriptive analyses for comparative outcomes. In addition, we conducted multivariable logistic regression analyses to assess the associations between the number of FABMs respondents clinics' have training on or offer materials for and respondents' knowledge, attitudes, and practices toward FABMs. All quantitative analyses were conducted using SAS version 9.4 (SAS Institute, Cary, NC).^9^

For open text, short-answer responses on questions about provider successes, and barriers to FABM provision, we conducted emergent thematic coding to identify existing themes and subthemes associated with each question. In addition, we conducted textual analyses on extension questions (*e.g.*, “other, please specify”) and quantitatively grouped these responses in new or existing answer categories.

## Results

An invitation to complete the survey was sent to 8002 e-mail addresses of family planning providers and was also placed on national websites as already detailed. A total of 458 family planning providers completed the anonymous online survey. This represents a 5.7% response rate of e-mailed invitations; however, with the inclusion of those who may have come across the survey on national websites, which is not possible to calculate, the response rate is likely much lower. The current sample includes 329 participants (71.8%) who met all inclusion criteria.

Of those completing the survey, 97.0% of respondents identified as female and almost half (45.0%) identified as clinical providers (NP, CNM, PA, MD, and DO), whereas another 48.0% identified as nurses. Among respondents who reported nursing licensure, the largest subspecialty were registered nurses (42.6%). Providers reported a variety of practice settings, including health departments (72.6%), community/federally qualified health centers (13.1%), and free-standing family planning organizations (8.5%) (Table 1).

**Table 1.** Participant and Clinic Demographics

Characteristic	N (%)
Gender	
Female	319 (97.0)
Male	4 (1.2)
Nonbinary/prefer to self-describe	4 (1.2)
Primary role	
Clinical provider (NP, CNM, PA, MD, and DO)	148 (45.0)
Registered nurse	140 (42.6)
Licensed vocational/practice nurse	17 (5.2)
Health educator/counselor/health care associate/medical assistant	16 (4.9)
Other	1 (0.3)
Principal setting	
Health department	239 (72.6)
Hospital-based setting	15 (4.6)
Planned parenthood	12 (3.6)
Free-standing family planning organization	28 (8.5)
Community health center/federally qualified health center	43 (13.1)
Tribal health center	8 (2.4)
Substance abuse treatment center	1 (0.3)
Faith-based organization/setting	1 (0.3)
Correctional facility-based setting	2 (0.6)
Federal government setting	1 (0.3)
Private foundation or nonprofit setting	6 (1.8)
Other	2 (0.6)
Location	
Urban	85 (25.8)
Suburban	50 (15.2)
Rural	182 (55.3)
Frontier	8 (2.4)
HHS region	
1. CT, ME, MA, NH, RI, VT	5 (1.5)
2. NJ, NY, Puerto Rico, Virgin Islands	15 (4.6)
3. DE, District of Columbia, MD, PA, VA, WV	17 (5.2)
4. AL, FL, GA, KY, MS, NC, SC, TN	113 (34.3)
5. IL, IN, MI, MN, OH, WI	25 (7.6)
6. AR, LA, NM, OK, TX	59 (17.9)
7. IA, KS, MO, NE	43 (13.1)
8. CO, MT, ND, SD, UT, WY	21 (6.4)
9. AZ, CA, HI, NV, American Samoa, Northern Mariana Islands, Micronesia, Guam, Marshall Islands, Palau	19 (5.8)
10. AK, ID, OR, WA	8 (2.4)
**Characteristic**	**Mean (SD)**
Average participant age	48.65 (11.7)
Average years since completing most advanced clinical training	15.6 (10.9)
Average years working at clinics/sites that provide family planning services	14.1 (11.3)
**Characteristic**	**Median (IQR)**
Median number of times per month counseled a person/couple on FABMs/NFP for pregnancy prevention	1 (10)

FABMs, fertility awareness-based methods; IQR, interquartile range; NFP, natural family planning; SD, standard deviation.

### Knowledge

A majority of participants answered all of the fertility questions correctly (52.0%). More than 90.0% of respondents could correctly identify both luteal phase length (91.2%) and characteristics of ovulatory cervical fluid (94.2%). Participants were less likely to know the typical length of the menstrual cycle (80.2%), or about the shift of basal body temperature after ovulation (74.5%).

Our multivariable regression model identified associations between the number of correct answers a participant reported and the reported number of different FABMs included in written information provided to patients (*β* = 0.192, *p* < 0.05) and by the number of FABMs that they reported formal training on (*β* = 0.195, *p* < 0.05) (Table 2).

**Table 2.** Regression Analyses

Predictor	N	Sum of FABMs included in written information provided to patients Beta or OR (CI)	Sum of FABMs those at facility have been formally trained on Beta or OR (CI)
Linear regression			
Belief of FABM viability	321	0.012	0.085
Knowledge score	329	0.192^*^	0.195^*^
Logistic regression			
Which of the following best describes your response when a patient asks you for information about FABMs for pregnancy prevention?			
Do not recommend FABMs	329	0.531 (0.232–1.213)	0.848 (0.455–1.58)
Calendar method	329	1.22 (1.04–1.44)^*^	1.05 (0.879–1.26)
Cervical mucus Method	329	1.54 (1.28–1.84)^***^	1.48 (1.19–1.85)^***^
Basal body Temperature method	329	1.41 (1.19–1.67)^***^	1.25 (1.03–1.50)^*^
Written information	329	1000 (0–1000)	1.47 (1.16–1.85)^**^
FABM mobile apps	329	1.55 (1.30–1.85)^***^	1.45 (1.18–1.77)^***^
Referral to location which specializes in FABM instruction	329	0.898 (0.718–1.122)	0.878 (0.664–1.16)
Referral to someone else in same office	329	0.270 (0.073–0.991)^*^	1.152 (0.822–1.62)
Other	329	0.142 (0.046–0.438)^***^	1.015 (0.776–1.33)

^*^ *p* < 0.05, ^**^p < 0.01, ^**^p < 0.001.

CI, confidence interval; OR, odds ratio.

### Attitudes

**FIG. 1.**
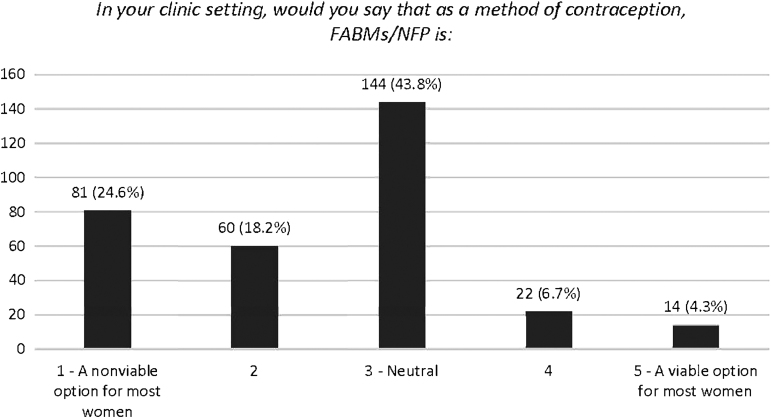
Attitudes toward FABMs. FABM, fertility awareness-based methods.

When asked about whether they thought FABMs were a viable option for contraception, most respondents gave an answer of “3” (43.8%) or “neutral,” with the second most popular answer being “1” (24.6%) or “a nonviable option for most women” (Fig. 1).

This response was not significantly impacted by the number of FABMs included in the written information provided to patients (*β* = 0.012, *p* = 0.778) or by the number of FABMs that those at the participant's facility have been formally trained on (*β* = 0.085, *p* = 0.087), although this number approaches significance.

### Clinical practices

When asked to identify how they responded to patient requests for FABMs for pregnancy prevention, providers generally responded by providing some information, either verbally (32.5%–48.0%) or through written materials (47.1%) (Table 3). Providers were significantly more likely to discuss a calendar method (odds ratio [OR] = 1.22, confidence interval [CI] 1.04–1.44), a cervical mucus method (OR = 1.54, CI 1.28–1.84), or a basal body temperature method (OR = 1.41, CI 1.19–1.67) with patients with each additional FABM added to the written materials available in the clinic. Providers were also significantly more likely to discuss a cervical mucus method (OR = 1.48, CI 1.19–1.85) or a basal body temperature method (OR = 1.25, CI 1.03–1.50) with each additional FABM that the clinic had formal training on.

**Table 3.** Clinical Practices

Characteristic	N (%)
Which of the following best describes your response when a patient asks you for information about FABMs for pregnancy prevention? (*n* = 329)	
I tell her they do not work and recommend something else	13 (4.0)
I describe the use of the calendar method	158 (48.0)
I describe the use of the cervical mucus method	125 (38.0)
I describe the use of the basal body temperature method	107 (32.5)
I provide her with written information on NFP/fertility awareness	155 (47.1)
I provide her with information about modern FABM mobile apps	98 (29.8)
I refer her to a location that specializes in FABM instruction	60 (18.2)
I refer her to someone else in our office	15 (4.6)
Other	40 (12.2)
What NFP/fertility awareness methods are included in the written information you provide patients? (*n* = 155)	
Symptothermal	32 (20.6)
Symptohormonal	12 (7.7)
Billings Ovulation Method	37 (23.9)
Two-Day Method	25 (16.1)
Standard Days	77 (49.7)
Natural Cycles	52 (33.5)
Lactational Amenorrhea Method	24 (15.5)
Other	11 (7.1)
Unknown	28 (18.1)
Does anyone at your facility have formal training in FABMs? (*n* = 329)	
Yes	78 (23.7)
What best describes the primary role of those at your facility who have formal training in FABMs? (*n* = 78)	
Manager/administrator	2 (2.6)
Clinical provider (NP, CNM, PA, MD, DO)	64 (82.1)
Registered nurse	28 (35.9)
Licensed vocational/practical nurse	5 (6.4)
Health educator/counselor/health care associate/medical assistant	13 (16.7)
Community outreach staff	3 (3.8)
Which FABMs have those at your facility been formally trained on? (*n* = 78)	
Symptothermal	15 (19.2)
Symptohormonal	5 (6.4)
Billings Ovulation Method	19 (24.4)
Two-Day Method	14 (17.9)
Standard Days	37 (47.4)
Natural Cycles	20 (25.6)
Lactational Amenorrhea Method	20 (25.6)
Other	5 (6.4)
Unknown	23 (29.5)

Seventy-eight (23.7%) participants reported someone at their facility had formal training in FABMs, the majority being clinical providers (82.0%). Almost half of those respondents reported training on the Standard Days method (47.4%). Respondents also reported receiving formal training on a symptothermal method (19.2%), symptohormonal methods (6.4%), Billings Ovulation method (24.4%), Two Day method (17.9%), Natural Cycles^®^ Mobile Application (25.6%), or the Lactational Amenorrhea method (25.6%) (Table 3).

### Qualitative responses

#### Barriers to providing FABMs as a contraceptive option

The most commonly reported barrier (*n* = 77) was provider perceptions that these methods were inappropriate for their clients. Other barriers to FABMs for pregnancy prevention included perceptions that clients did not desire to use FABMs, that clients could not afford the supplies required to use an FABM, and that FABMs themselves are less effective methods of contraception compared with other methods (Table 4).

**Table 4.** Qualitative Findings

Examples of reported provider barriers to providing FABMs (n = 226)
Provider perceptions that these methods are inappropriate for their clients (*n* = 77)
“[We] have poorly educated patients who don't know basic anatomy or body functions. They have hard enough time making it to their appointment due to lack of resources and poor schedules.” Female provider, age 41–45 years, Missouri
“Many patients do not seem to have the self-control or motivation to be strict within the guidelines of abstinent/condom times.” Female provider, age 61–65 years, Indiana
“We also have many women with irregular menses and partners that do not agree that women get to determine when sexual contact occurs. In other words, the male partner determines when the couple has sex, not the female, therefore planned sexual contact is not possible.” Female provider, age 36–40 years, North Carolina
“Most of our patients who are seeking contraception want a method which is more reliable. We have a lot of teenagers, and college students who have busy lives and do not want to worry about their contraception. They would rather have a LARC. Also, if they are coming in for contraception, they are planning on leaving with a method, not a calendar.” Female provider, age 36–40 years, New York
Provider perceptions that FABMs are ineffective (*n* = 15)
“I have no challenge about the method if someone chooses to use it. I have both a niece and nephew who were very much unplanned using this method.” Female registered nurse, age 46–50 years, Louisiana
“There are other methods with more demonstrated effectiveness to prevent pregnancy.” Female provider, age 55–60 years, Tennessee
“I consider it valuable at the bottom of the preferred option list—better than simple withdrawal and am biased as to the value of more effective methods for most women.” Female provider, age 51–55 years, Pennsylvania
Provider education and training barriers (*n* = 33)
“Lack of education. I don't feel I have enough training to give accurate information on all the different NFP methods.” Female registered nurse, age 46–50 years, Montana
“There is also no one here who has had formal training…although I would love to!” Female provider, age 26–30 years, North Dakota
“We have educational material to hand out to the patients, however no one in the facility is certified or specially trained on FABMs.” Female provider, age 51–55 years, Texas
Provider time and scheduling barriers (*n* = 27)
“No time for education because I have patients every 15 minutes and we get overbooks and walk-ins and can't say no to late patients” Female provider, age 41–45 years, Minnesota
“Counseling takes more time than other methods.” Female provider, age 61–65 years, Pennsylvania
“Time restraint during busy clinic to fully explain.” Female registered nurse, age 36–40 years, Louisiana
Providers administrative/funding barriers (*n* = 9)
“It is not a tier 2 or tier 3 method of contraception. It is not recommended under Title X as a preferred pregnancy prevention method. We do not have any written documents explaining FABMs. There are no educational tools to show visual or kinesthetic learners.” Female health educator, age 21–25 years, California
“Systems issues which do not support; FP funders will not accept this as a method in our EMR” Female provider, age 66–70 years, New Jersey
Perception of a lack of demand for FABMs (*n* = 21)
“We have so many other more effective methods that patients aren't usually interested in FABMs.” Female provider, age 56–60 years, Colorado
“Our clinic sees many clients under the age of 19. When reviewing this as a preventive service, they are not interested in taking their temperature, checking mucus, *etc.* Many report that it is easier to take a pill.” Female registered nurse, age 46–50 years, Illinois
“Patients come to us for a method of birth control, not about how to prevent pregnancy using FABMs.” Female registered nurse, age 56–60 years, Nevada
**Examples of reported provider successes with providing FABMs for pregnancy prevention (*n* = 200)**
No successes (*n* = 72)
“We have had very little success. In fact, we have had unintended and unwanted pregnancies as a result. Stressing this as a truly effective and viable option for women is, in my personal opinion, taking a huge step backward and I am disturbed to see the renewed focus on suggesting this as a ‘good’ option for women, particularly for adolescents who already struggle with impulse control. Fertility awareness is the reason I and 3 of my 4 brothers and sisters are here.” Female provider, age 56–60 years, North Carolina
“I personally do not feel that I have had any successes with this method and frankly feel that this is a method best used by monogamous individuals and families seeking pregnancy rather than pregnancy prevention.” Female provider, age 61–65 years, Missouri
“None! Title X is going to be worthless now.” Female provider, age 41–45 years, Minnesota
Not sure or no follow-up on FABM use among women counseled (*n* = 20)
“I make the referral, but do not know the outcome.” Female provider, age 50–55 years, North Carolina
“I have only had two ask about it, as they did not want any hormones. I have not seen them since their original evaluation. I am unsure if they information we gave them helped.” Female provider, age 50–55 years, Iowa
Successful among certain “types” of women (*n* = 12)
“Where [I have seen success] has been in more traditional communities with less frequent intercourse and supporting other traditions of abstinence.” Community outreach individual, Washington
“People usually want something more reliable but it is great for ‘refugees’ or people who want no method and no further children.” Female provider, age 66–70 years, Virginia
Success in improving body literacy (*n* = 10)
“I have had a few patients looking specifically for a FAM. The discussion increases awareness of their bodies and how they work which is always fabulous!” Female provider, age 41–45 years, Arizona
Patient satisfaction when utilized (*n* = 7)
“Nearly everyone who chooses it, uses it for a long time (much longer than hormonal methods other than IUDs and implant).” Female health counselor, age 46–50 years, California
“The few that follow this method are satisfied.” Female registered nurse, age 31–35 years, Louisiana

Many providers identified that they and their clinical staff did not have the training to counsel on these methods. They also noted the lack of available educational materials to give to women interested in learning about FABMs. Many spoke about the time-intensive nature of counseling on these methods and the struggle to fit this type of discussion into a 15-minute visit window. Others noted funding and administrative limitations to inclusion of these methods in counseling such as a lack of funder reimbursement for these methods, or a lack of administrative support to develop educational materials or train staff on FABMs.

#### Successes in providing FABMs as a contraceptive option

The majority of providers did not report any successes in incorporating FABMs into their contraceptive counseling for pregnancy prevention. Those who had successes associated them with certain demographic groups of women who used them. Successes mentioned with incorporating FABMs in contraceptive counseling included successful use to avoid pregnancy in conjunction with barrier methods and/or mobile apps such as Cycle Beads (Standard Days method), improved body literacy and fertility knowledge, and good outcomes with high satisfaction among women who were “self-motivated” (Table 4).

## Discussion

In our knowledge, this is the first study to assess provider attitudes, knowledge, and behaviors on FABMs within the Title X network of providers in the United States. The low response rate should be noted and data treated as a preliminary study. The findings of this study may not represent the views of the entire Title X network of providers, especially as we think those who feel passionately about the topic (either positively or negatively) were more likely to respond than those who felt more neutral. It is also important to note that although FABMs are used widely internationally, this study is not representative of any global populations.

Although a majority of providers had correct general knowledge of fertility, few providers demonstrated positive attitudes about offering FABMs as a viable option for pregnancy prevention. Most reported neutral or negative perceptions about these methods, something that has been found in other studies,^10,11^ coupled with a common perception of these methods as “ineffective” or inappropriate for their clients. This finding is similar to that found in previous studies in non-Title X providers, where a majority of clinicians underestimate the effectiveness of FABMs and do not always provide all information about modern FABMs.^6,7,10,11^

We found preliminary evidence to suggest that providers who had written materials around multiple methods of FABMs were more likely to counsel clients around FABM use. This may be due to increased confidence among providers who had received resources to provide these methods or a better understanding of FABM effectiveness data, but it could also be that providers who were more likely to counsel about FABM use were also more likely to have written materials about their use.

Our qualitative analyses identified significant provider barriers related to offering FABMs as a viable option for specific client populations, including those with low health literacy, individuals who are single parenting, young people, and those who are facing challenging life circumstances. Patients with low health literacy may incorrectly identify their fertile window,^12^ which is an opportunity for reproductive education interventions. FABMs cannot be used effectively and should not be recommended in a relationship in which timing of intercourse cannot be mutually decided upon and/or a barrier method cannot be used. For women who have unique reproductive considerations (*e.g.*, long cycles, anovulation, and breastfeeding), there is limited data about FABM effectiveness. However, even in some of these cases, an FABM may be the only method that a person would consider, for example, if they have a religious consideration. Therefore, even these groups deserve to have a reliable source of information about FABMs. Perhaps more importantly, FABMs have been used with typical use pregnancy rates similar to those for hormonal methods and barrier methods in several populations that were a point of concern among participants, including low-literacy populations^13–20^ and young single women with multiple partners.^21–24^ One small study in the midwest indicated that when women were educated about FABMs, more were interested in using them.^6^ Therefore, some of these provider barriers may reflect misperceptions of the effectiveness of FABMs that could be addressed through increased education and training, as well as improved clinic materials.

FABM education for clinicians or patients should stress the differences between the many modern methods. There are methods available that are designed to be simple and easy to use with little or no equipment or supplies needed, some are complex and require some equipment, supplies, or technology, and all methods differ in their effectiveness of pregnancy prevention.^1^ Some methods are not suitable for women with irregular menstrual cycles and some women may have preferences on the symptoms they check. All these aspects of the different FABMs should be taken into consideration when providing contraceptive counseling to a patient who is interested in FABMs. In addition, the differences between methods may dispel some beliefs that patients or providers have about FABMs if they view them as a whole, such as the belief that they are not effective or the belief that they require too much equipment.

Clinicians reported providing verbal and written information to clients who requested FABMs; however, few clinics reported having training or education around FABMs onsite. Other studies have shown that medical students or residents are unlikely to include FABMs in curriculum or learning experiences.^5,25,26^ Several providers noted a lack of time for FABM counseling within existing clinical schedules. This barrier mirrors a similar reported time barrier for other contraceptive services, including same-day insertions of intrauterine devices or vasectomy,^27^ and methods such as Standard Days Method can be provided within the typical visit time frame, particularly if written materials are also available. Opportunities for improvement may include the development of FABM clinical trainings as CME-approved offerings, as providers are more engaged in learning new methods when they support their licensure requirements. In addition, the development of comprehensive written materials that provide an overview of multiple methods may improve both provider self-efficacy and increase clinical knowledge. Recent studies have found that provider method bias is common,^28,29^ but additional training on contraceptive shared decision making may be an opportunity to reduce bias against certain methods, such as FABMs.^30^ Table 5 includes some links to training and resources for clinicians on FABMs.

**Table 5.** Educational Resources for Fertility Awareness-Based Methods

Overviews on FABMs for clinicians
• Contraceptive Technology 21st Ed
○ http://www.contraceptivetechnology.org/the-book/
• World Health Organization's medical eligibility criteria for contraceptive use, 5^th^ Ed
○ https://www.who.int/reproductivehealth/publications/family_planning/MEC-5/en/
• Family Planning Handbook: A Global Handbook for Providers, ch 18, 2018
○ http://fphandbook.org/sites/default/files/global-handbook-2018-full-web.pdf
• Free webinars from the National Clinical Training Center for Family Planning, supported by the U.S. Office of Population Affairs Title X Family Planning Program, CE available
○ Understanding and Counseling Potential Users on Fertility Awareness Based Methods for Pregnancy Prevention
▪ https://vimeo.com/264114233
○ Effectiveness of Fertility Awareness Based Methods for Pregnancy Prevention
▪ https://vimeo.com/284453322
○ Fertility Apps: A New Approach for Fertility Awareness Based Methods
▪ https://vimeo.com/277724852
**Training for clinicians**
• Standard Days method
○ Online, free 1–2 hours training module, CME available
○ http://archive.irh.org/SDM_Training/index.php
• Two-Day Method
○ Overview and resources available
○ http://irh.org/twoday-method/
• Sensiplan (symptothermal method)
○ Materials and training available at cost
▪ https://www.sensiplan-im-netz.de/?page_id=904
○ In-person teacher training at Reply Ob/Gyn in 2021
▪ Contact lhartley@replyobgyn.com
○ Sensiplan user manual and workbook available at cost
▪ https://replyobgyn.com/store/product/sensiplan-physical-bundle/
• Billings Ovulation Method
○ Religious components, teacher training available at cost
○ https://www.woombinternational.org/education/correspondence-course
• Marquette Symtohormonal Method
○ Religious components, teacher training available at cost
○ https://www.marquette.edu/nursing/natural-family-planning-teacher.php

Additional research is needed to better understand the acceptability of FABMs as contraception for both providers and patients and determine best practices in offering and counseling on FABMs in a family planning visit. Assessing the success of clinical interventions to improve scheduling availability for same-day services that include FABMs would provide valuable insight into how these methods can be practically incorporated into the clinic offering. Other important areas of future study include demonstrating whether broader understanding of fertility information may be helpful in health decision making and assessing whether emerging digital fertility applications lead to increased options for those who desire to use FABMs.

Patient satisfaction and successful family planning outcomes have been directly tied to increased availability of the full range of contraceptive methods.^31^ Incorporating FABMs into contraceptive counseling represents an opportunity to increase the contraceptive offering for clients who want them. Despite existing barriers to clinical provision, increasing demand for these methods, as well as recent federal interest in their availability, could support the development of new strategies to incorporate FABMs more fully into contraceptive counseling.

## Abbreviations Used

CI: confidence interval
FABMs: fertility awareness-based methods
NFP: natural family planning
OR: odds ratio

## Appendix A1

### Appendix A1. Survey Questionnaire

1. Does the clinic/service site where you work most of the time (primary clinic setting) provide family planning services?
2. () Yes
3. () No
2. Does your primary clinic setting receive federal Title X family planning funds?
5. () Yes
6. () No
7. () Not sure
3. Where is your primary clinic setting located? (state or territory)
4. What is your age? (years)
5. What is your current gender identity?
11. () Female
12. () Male
13. () Transgender male/trans man
14. () Transgender female/trans woman
15. () Nonbinary, gender nonconforming, genderqueer, genderless, or two-spirit
16. () Prefer to self-describe
6. What best describes your primary role at your primary workplace/family planning setting?
18. () Manager/administrator
19. () Clinical provider (NP, CNM, PA, MD, DO)
20. () Registered nurse
21. () Licensed vocational/practical nurse
22. () Health educator/counselor/health care associate/medical assistant
23. () Community outreach staff
24. () Billing/finance assistant
25. () Other
7. What is your specialty/area of practice?
27. [] Adult medicine/internal medicine
28. [] Family practice/family medicine
29. [] Infectious disease
30. [] Midwifery
31. [] Ob/gyn
32. [] Women's health
33. [] Pediatrics
34. [] Other
8. How many years has it been since you completed your most advanced clinical training (residency, fellowship, *etc.*)?
9. How many years total have you been working at clinics/sites that provide family planning services?
10. What are the principal settings in which you provide family planning services?
38. [] Health department (*e.g.*, state, county, local)
39. [] Hospital-based setting
40. [] Planned parenthood
41. [] Free-standing family planning organization
42. [] Community health center/federally qualified health center
43. [] Tribal health center
44. [] University- or school-based setting
45. [] Substance abuse treatment center
46. [] Faith-based organization/setting
47. [] Correctional facility-based setting
48. [] Federal government setting
49. [] Private foundation or nonprofit setting
50. [] Other
11. Describe the area where your primary practice site/clinic is located:
52. () Urban
53. () Suburban
54. () Rural
55. () Frontier
12. On average, how many patients do you see for family planning services each week?
57. *The correct answer for the knowledge questions below is denoted with an asterisk
13. Normal menstrual cycles are between 21 and 38 days in length.
59. () True*
60. () False
14. Ovulation is usually preceded by clear stretchy vaginal discharge.
62. () True*
63. () False
15. Certain sexual positions can increase one's chance of getting pregnant.
65. () True
66. () False*
16. Ideally, intercourse should occur the day before or the day of ovulation for fertilization to occur.
68. () True*
69. () False
17. Having intercourse more than once per day will increase the chance of conception.
71. () True
72. () False*
18. Basal body temperature rises about half a degree Fahrenheit after ovulation.
74. () True*
75. () False
19. Having intercourse three or more days after an increase in basal body temperature increases the chance of conception.
77. () True
78. () False*
20. Ovulation typically occurs ∼12–16 days before menses.
80. () True*
81. () False
21. Peak fertility for women occurs at what age?
83. () 23*
84. () 30
85. () 35
86. () 40
22. What is the best predictor of difficulty in conceiving?
88. () Age >35 years*
89. () Low anti-Mullerian hormone level
90. () High follicle stimulating level
91. () Body mass index 30 kg/m^2^ or higher
23. Which of the following factors can increase fertility?
93. () Use of water-soluble sexual lubricant
94. () Living in a rural instead of urban environment
95. () Lying down for 10 minutes after intercourse
96. () None of the above*
24. Which of these factors places the woman at highest risk for infertility?
98. () Being >35 years*
99. () Being under a lot of stress
100. () Smoking >10 cigarettes per day
101. () Having more than two alcoholic drinks per day
25. A 28-year-old woman desiring pregnancy should consult a fertility/infertility specialist after a failure to become pregnant after how many months of unprotected vaginal intercourse?
103. () 3 months
104. () 6 months
105. () 12 months*
106. () 24 months
26. A 38-year-old woman desiring pregnancy should consult a fertility/infertility specialist after a failure to become pregnant after how many months of unprotected vaginal intercourse?
108. () 3 months
109. () 6 months*
110. () 12 months
111. () 24 months
27. How many times in the past month have you counseled a person/couple on FABMs/NFP planning for pregnancy prevention?
28. How many times in the past month have you counseled a person/couple on FABMs/NFP to attempt to achieve pregnancy?
29. Which of the following best describes your response when a patient asks you for information about FABMs for pregnancy prevention?
115. [] I tell her they do not work and recommend something else
116. [] I describe the use of the calendar method
117. [] I describe the use of the cervical mucus method
118. [] I describe the use of the basal body temperature method
119. [] I provide her with written information on NFP/fertility awareness
120. a. What NFP/fertility awareness methods are included in the written information you provide patients?
121. [] Symptothermal (Sensiplan)
122. [] Symptohormonal (Marquette)
123. [] The Billings Ovulation Method
124. [] Two-Day Method
125. [] Standard Days (Cycle Beads)
126. [] Natural Cycles
127. [] LAM (Lactational Amenorrhea Method)
128. [] Other
129. [] Unknown
130. [] I provide her with information about modern FABM mobile applications
131. b. Which mobile apps do you recommend patients?
132. [] I refer her to a location that specializes in FABM instruction
133. [] Other
30. Which of the following do you usually recommend as initial steps for a woman/couple who are having difficulty achieving pregnancy, do not meet the definition of “infertility” and have had no previous evaluation for infertility?
135. [] Intercourse at least every other day during the entire cycle
136. [] Intercourse during midcycle (*i.e.*, cycle day 10–16)
137. [] Observation of cervical mucus and directed intercourse to days with high-quality (fertile) mucus and a few days after
138. [] Observation of basal body temperature and directed intercourse in the days leading up until the time of the temperature increase
139. [] Urine-based over-the-counter LH surge/ovulation test kits and intercourse on the day of the LH surge
140. [] Following advice from menstrual cycle tracking or fertility tracking mobile applications
141. a. Please specify which specific mobile apps you recommend.
142. [] Other
31. Does anyone at your facility have formal training in FABMs?
144. () Yes
145. a. What best describes the primary role of those at your facility who have formal training in FABMs?
146. [] Manager/administrator
147. [] Clinical provider (NP, CNM, PA, MD, and DO)
148. [] Registered nurse
149. [] Licensed vocational/practical nurse
150. [] Health educator/counselor/health care associate/medical assistant
151. [] Community outreach staff
152. [] Billing/finance assistant
153. [] Other
154. b. Which FABMs have those at your facility been formally trained on?
155. [] Symptothermal (Sensiplan)
156. [] Symptohormonal (Marquette)
157. [] The Billings Ovulation Method
158. [] Two-Day Method
159. [] Standard Days (Cycle Beads)
160. [] Natural Cycles
161. [] LAM (Lactational Amenorrhea Method)
162. [] Other
163. [] Unknown
164. () No
32. In your clinic setting, would you say that as a method of contraception, FABMs/NFP is:
166. () 1—A nonviable option for most women
167. () 2
168. () 3—Neutral
169. () 4
170. () 5—A viable option for most women
33. Tell us about your personal or your clinic's challenges with offering FABM as a method of preventing pregnancy:
34. Tell us about your personal or your clinic's challenges with offering FABM as a method of achieving pregnancy:
35. Tell us about your personal or your clinic's successes with offering FABM as a method of preventing pregnancy:
36. Tell us about your personal or your clinic's successes with offering FABM as a method of achieving pregnancy.
